# Association of Bone Metabolism Indices and Bone Mineral Density with Diabetic Retinopathy in Elderly Patients with Type 2 Diabetes Mellitus: A Cross-Sectional Inpatient Study in China

**DOI:** 10.1155/2021/8853622

**Published:** 2021-01-12

**Authors:** Xin Zhao, Lili Huo, Xiaofeng Yu, Xiaomei Zhang

**Affiliations:** ^1^Endocrinology Department, Peking University International Hospital, Beijing, China; ^2^Endocrinology Department, Beijing Jishuitan Hospital, Beijing, China

## Abstract

**Objective:**

This study is aimed at analyzing the association between bone metabolism indices and diabetic retinopathy (DR) in elderly patients with type 2 diabetes mellitus.

**Methods:**

Data of 352 men and 284 postmenopausal women, aged more than 50 years, with type 2 diabetes mellitus were retrospectively analyzed. Patients were divided into three groups based on the degree of DR: nondiabetic retinopathy (NDR) group, background diabetic retinopathy (BDR) group, and proliferative diabetic retinopathy (PDR) group.

**Results:**

(1) The diabetic duration and urinary albumin to creatinine ratio (UACR) were significantly higher in the PDR and BDR groups than in the NDR group (*P* < 0.05). The level of beta-C-terminal telopeptide (*β*-CTX) in male patients was lower in the PDR and BDR groups than in the NDR group (*P* < 0.05). In addition, the level of procollagen 1 intact N-terminal (P1NP) in female patients was higher in the PDR and BDR groups than in the NDR group (*P* < 0.05). (2) For men and postmenopausal women, the proportion of vitamin deficiency was higher in the PDR and BDR groups than in the NDR group (*P* < 0.05). (3) The logistic regression analysis in men and postmenopausal women showed that the diabetic duration and lower levels of UACR and 25(OH)D were independent risk factors for DR (*P* < 0.05). (4) The diabetic duration was also an independent risk factor for PDR (*P* < 0.05); however, no independent correlation was found between the level of 25(OH)D and PDR (*P* > 0.05).

**Conclusions:**

A close association was observed between 25(OH)D level and DR in the elderly male patients and postmenopausal women with type 2 diabetes mellitus. P1NP and *β*-CTX levels might be closely related to DR in elderly male patients and postmenopausal women with type 2 diabetes mellitus.

## 1. Introduction

An increasing number of people have been diagnosed with type 2 diabetes mellitus (T2DM) with social and economic development. Diabetes affects all systems of the body and is followed by many complications. Diabetic retinopathy (DR) is a common microvascular complication of T2DM. Recent clinical studies have pointed out an acceleration in the occurrence and development of DR. DR often presents with blurred and diminished vision; however, it is difficult to be detected in the early stage. The progression of DR is expected to rise further or even lead to blindness in patients with poor blood glucose control [1].

Previous studies have shown that vitamin D deficiency is an independent risk factor for DR in patients with type 1 diabetes mellitus, and bone metabolism may be closely related to retinopathy [2]. However, whether this relationship also exists in patients with T2DM is still controversial [3, 4] because most prior studies focused on postmenopausal women [5]. The relationship of bone metabolism indices and bone mineral density with DR is currently under exploration. Identifying any potential correlation between bone metabolism and DR in patients with T2DM, as early as possible, has great significance in the prevention and treatment of DR and may have other related clinical applications.

The purpose of this study was to explore the correlation of DR with bone metabolism indices by comparing the changes in bone metabolism indices and bone mineral density between DR and nondiabetic retinopathy (NDR), providing new evidence for preventing and treating DR and related clinical manifestations.

## 2. Participants and Methods

### 2.1. Participants

This retrospective study was performed on 352 men and 284 postmenopausal women, aged more than 50 years, with T2DM who were consecutively hospitalized at the endocrinology department of Peking University International Hospital from July 2017 to September 2019. The average age of men and women was 62.54 ± 8.14 years and 63.02 ± 8.30 years, respectively, and the average duration of T2DM was 10.92 ± 7.94 years. All participants met the T2DM diagnostic criteria of the World Health Organization in 1999 [6]. The exclusion criteria were as follows: patients with (1) other types of diabetes mellitus; (2) nonphysiological menopause; (3) a history of long-term drug use that affected bone metabolism; (4) a history of primary or secondary bone cancer; (5) a history of drug use (estrogen, bisphosphonate, active vitamin D, etc.) for osteoporosis (OP).

## 3. Methods

### 3.1. Baseline Characteristics

The baseline characteristics were as follows:

(1) *Basic Information Collection*. Details on age, date of birth, diabetic duration, and chronic history of all patients were collected and recorded

(2) *Height and Weight Measurement*. All the participants were instructed to take off their shoes and socks and wear light clothing, following which height (cm) and weight (kg) of each participant were measured. Body mass index (BMI) was acquired using the following formula: weight/height^2^ (kg/m^2^). Blood pressure, including systolic blood pressure (DBP) and diastolic blood pressure (SBP), was measured in all participants

### 3.2. Laboratory Measurements

Venous blood samples were collected from all participants after at least 8 h of fasting in the morning. Laboratory measurements included fasting blood glucose (FBG), serum creatinine (sCr), glycosylated hemoglobin (HbA1c), calcium (Ca), uric acid (UA), low-density lipoprotein cholesterol (LDL-C), total cholesterol (TC), triglyceride (TG), high-density lipoprotein cholesterol (HDL-C), parathyroid hormone (PTH), osteocalcin (OC), beta C-terminal telopeptide (*β*-CTX), procollagen 1 intact N-terminal (P1NP), and 25-hydroxyvitamin D [25(OH)D]. Urine was collected from all the participants on three consecutive mornings, and urinary albumin to creatinine ratio (UACR) values were calculated using the immunoturbidimetric method. The estimated glomerular filtration rates (eGFRs) were determined using the CKD-EPI-ASIA equation. According to the current recommendations of most international and domestic institutions and experts [7], a value of 25(OH)D < 20 ng/mL indicated vitamin D deficiency.

### 3.3. Bone Mineral Density Measurement

The bone mineral density (BMD) of the hip and lumbar spine of the participants was measured using dual-energy X-ray absorptiometry (DXA) [8], which automatically generated the *T* value based on the BMD using a software (Hologic, USA) in the laboratory of Peking University International Hospital. *T* value = SD of (BMD-BMD of the normal people with the same race and sex)/BMD of the normal people with the same race and sex. According to WHO standard [9], *T* value > -1.0 SD is normal BMD; -2.5 SD ≤ *T* value ≤ -1.0 SD is osteopenia; *T* value < -2.5 SD is osteoporosis.

## 4. Diabetic Retinoscopy

Fundus photography based on the staging standard of DR was routinely performed on each participant in the hospital [10]. The following staging criteria were used: stage 1, a small bleeding spot or microangioma noted; stage 2, a hard exudation found; stage 3, a soft cotton-like exudation noted; stage 4, blood accumulation and neovascularization of retinal vitreous found; stage 5, proliferation of fibrous blood vessels and subsequent vitreous organization noted; and stage 6, traction retinal detachment found leading to blindness. Among these, stages 1–3 were classified as background retinopathy (BDR), while stages 4–6 were classified as proliferative retinopathy (PDR). According to the degree of retinopathy, the patients were divided into three groups: NDR group, BDR group, and PDR group.

## 5. Statistical Analysis

All data were processed using SPSS 25.0. Normally distributed data were shown as average ± standard deviation (*x* ± *s*), while nonnormally distributed data were shown as median and quartile spacing. Variance analysis was used for intergroup comparisons. The unit of count is expressed by rate, and the chi-square test was used for intergroup comparisons. The logistic regression method was used to analyze the main influencing factors for DR. *P* < 0.05 indicated a statistically significant difference.

## 6. Results

### 6.1. Comparison of General Characteristics, Biochemical Indices, BMD, and Bone Metabolism Indices among NDR, BDR, and PDR Groups

The diabetic duration was significantly higher in the PDR and BDR groups than in the NDR group among elderly male patients with T2DM (*F* = 10.95, *P* < 0.05). The diabetic duration was higher in the PDR group than in the BDR group (*P* < 0.05). Similarly, the UACR level was significantly higher in the PDR and BDR groups than in the NDR group (*F* = 7.18, *P* < 0.05); the level in the PDR group was significantly higher than that in the BDR group (*P* < 0.05). Also, significant differences were found in the levels of *β*-CTX and 25(OH)D among the three groups (*P* < 0.05). The levels of *β*-CTX and 25(OH)D were lower in the PDR group than in the other two groups; the levels in the BDR group were significantly lower than those in the NDR group (*P* < 0.05). No significant difference was observed in blood glucose and blood lipid levels, blood pressure, BMD, and PTH level among the three groups (*P* > 0.05).

For postmenopausal women with T2DM, the diabetic duration was significantly higher in the PDR and BDR groups than in the NDR group (*F* = 13.96, *P* < 0.05); the duration in the PDR group was significantly higher than that in the BDR group (*P* < 0.05). Similarly, the UACR level was significantly higher in the PDR and BDR groups than in the NDR group (*F* = 6.35, *P* < 0.05); the level in the PDR group was significantly higher than that in the BDR group (*P* < 0.05). In postmenopausal women with T2DM, a significant difference was noted in the levels of P1NP and 25(OH)D among the three groups (*P* < 0.05). The level of 25(OH)D was lower in the PDR group than in the other two groups, with the level of 25(OH)D in the BDR group being significantly lower than in the NDR group (*P* > 0.05). Similarly, the level of P1NP was higher in the PDR group than in the other two groups, with the level of P1NP being significantly higher in the BDR group than in the NDR group (*P* < 0.05). No significant difference was found in blood glucose, TC, TG, LDL-C, and HDL-C levels; blood pressure; BMD; PTH level; and other indicators among the three groups (*P* > 0.05) (Table 1).

### 6.2. Relationship between Vitamin D Status and DR

For elderly male patients, the prevalence of vitamin D deficiency in the PDR group was 87.5%, which was higher than that in the BDR group. A significant difference was observed among the three groups (*χ*^2^ = 7.75, *P* < 0.05). For postmenopausal women, the prevalence of vitamin D deficiency in the PDR group was 84.6%, which was higher than that in the BDR and PDR groups (*χ*^2^ = 7.51, *P* < 0.05) (Table 2).

### 6.3. Analysis of Factors Influencing DR

For elderly male patients, the occurrence of DR was considered as the dependent variable, and a statistically significant index from single-factor analysis was taken as the independent variable for logistic regression analysis. The results showed that diabetic duration, higher level of UACR, and lower levels of *β*-CTX and 25(OH)D were the risk factors for DR (*P* < 0.05). After adjusting for age; blood pressure; blood glucose, TC, TG, LDL-C, and HDL-C levels; and other factors, the diabetic duration, higher UACR level, and lower 25(OH)D level were found to be independent risk factors for DR (*P* < 0.05).

For postmenopausal women, the occurrence of DR was considered as the dependent variable, and the statistically significant index from single-factor analysis was taken as the independent variable for logistic regression analysis. The results showed that diabetic duration, higher levels of UACR and PINP, and lower levels of 25(OH)D were the risk factors for DR (*P* < 0.05). After adjusting for age, blood glucose level, blood pressure, blood lipid level, and other factors, the diabetic duration, lower UACR level, and lower 25(OH)D level were found to be independent risk factors for DR (*P* < 0.05) (Table 3).

### 6.4. Analysis of Factors Influencing PDR

For elderly male patients with DR, only diabetic duration was found to be the risk factor for PDR after adjusting for age, blood glucose level, blood pressure, blood lipid level, and other factors (OR = 1.03, *P* < 0.05).

For postmenopausal female patients with DR, only diabetic duration was found to be the risk factor for PDR after adjusting for age, blood glucose level, blood pressure, blood lipid level, and other factors (OR = 1.13, *P* < 0.05) (Table 4).

## 7. Discussion

The number of people suffering from DR has increased rapidly with an increase in the prevalence of diabetes [11, 12]. DR has become a common diabetes-associated complication. Some studies have indicated that 0.2%–0.5% of patients with diabetes may become blind [13]. DR has a serious impact on human health and quality of life, making it a serious concern worldwide. To solve this problem, a lot of research has been done on DR [14, 15].

Considering the high incidence rate of DR, early exploration of the possible risk factors for DR is important, as this may significantly delay disease progression. Recent studies have found that the bone metabolism indices and vitamin D are related to DR, thus, providing a new way for finding treatment options for DR.

Vitamin D has a wide range of functions because its receptor is expressed in various tissues such as the pancreas. Researchers have found that some vitamin D receptor genes have a protective effect on the retina, while others are harmful to the diabetic retina because vitamin D receptor gene polymorphisms are related to the occurrence of DR [16]. Knocking down harmful genes or overexpressing protective genes may reduce the incidence of DR. Zoppini et al. [17] examined the relationship between vitamin D and diabetic microangiopathy by analyzing the results of 25(OH)D and fundus examination in 715 patients with T2DM. They found that the lower the level of 25(OH)D, the more severe the DR, suggesting that 25(OH)D was independently related to DR. Similarly, Bang et al. [5] analyzed the studies before 2016, including 17,664 people from 15 studies, and found a 1.03-time increase in the risk of DR in patients with vitamin D deficiency.

The present study found that the 25(OH)D level in elderly male patients and postmenopausal women decreased statistically significantly in the BDR and PDR groups. The proportion of patients with vitamin D deficiency in DR was relatively higher. The result of logistic regression analysis also indicated that the lower level of 25(OH)D might be a risk factor for DR in both male and female patients. However, further analysis revealed that the level of 25(OH)D in elderly men and postmenopausal women was significantly higher in the BDR group than in the PDR group among patients diagnosed with DR. However, the 25(OH)D level was not found to be a protective factor for BDR after adjusting for age; diabetic duration; blood glucose, TC, TG, LDL-C, and HDL-C levels; blood pressure; eGFR; and other factors.

Some studies have shown that the rate of bone formation and absorption is consistent with the degree of DR. Bone metabolism also shows a high conversion state in microvascular disease. The rate of bone conversion also accelerates accordingly with the aggravation of microvascular disease as seen in DR, and the rate of bone absorption is significantly faster than the rate of bone formation, suggesting that bone metabolism indices may reflect an underlying microvascular disease.

The nutritional supply to bone tissue is compromised because of the damage to microcirculation in the periosteum, causing inadequate proliferation and differentiation of bone marrow stromal cells, which eventually are depleted. As a result, the bone nerves are damaged and the metabolism of bone tissue is abnormal. Therefore, the bone metabolism indices can reflect these earlier changes in microcirculation. A study investigating the changes in the bone metabolism indices in patients with T2DM reported that the index was different compared with in healthy patients [18–20] and the CTX, NTX, P1NP, and OC levels in patients with T2DM reduced. The study proposed that the bone metabolism indices in patients with T2DM could truly reflect the biological effects on bone in such patients. Other studies explored the relationship between bone metabolism indices and complications of patients with T2DM. A previous cross-sectional study [21] showed that the bone metabolism indices could change before BMD in patients with early diabetic nephropathy, revealing the relationship between bone metabolism indices and diabetic microangiopathy in the population.

In view of the different characteristics of bone metabolism indices based on sex, this study compared the bone metabolism indices between elderly men and postmenopausal women with T2DM. First, in the elderly male patients, the level of *β*-CTX showed a significant decrease in the BDR and PDR groups. The logistic regression analysis also showed that the lower level of *β*-CTX could be associated with the occurrence of DR. However, the decrease in the level of *β*-CTX was not found to be an independent risk factor for DR after adjusting for age; diabetic duration; blood glucose, TC, TG, LDL-C, and HDL-C levels; blood pressure; eGFR; and other factors. In addition, the decrease in the level of *β*-CTX was not found to be an independent risk factor for PDR after adjusting for age; diabetic duration; blood glucose, TC, TG, LDL-C, and HDL-C levels; blood pressure; eGFR; and other factors. For postmenopausal women, the results showed that the level of P1NP increased significantly in the BDR and PDR groups compared with the NDR group. The logistic regression analysis also showed that the increase in the level of P1NP could be associated with the occurrence of DR, but the level of P1NP was not found to be an independent risk factor for DR after adjusting for age; diabetic duration; blood glucose, TC, TG, LDL-C, and HDL-C levels; blood pressure; eGFR; and other factors. The level of P1NP was also not found to be an independent risk factor for PDR after adjusting for age, diabetic duration, blood glucose, blood lipid, blood pressure, eGFR, and other factors.

In this study, no significant difference in blood lipid level was found among the NDR, BDR, and PDR groups. Studies in China and abroad explored the impact of blood lipid level on DR, but the results were inconclusive [22–24]. This study found no significant correlation between blood lipid level and DR; however, the results might have been affected by factors such as small sample size, no statistical use of lipid-lowering drugs, no follow-up, and so forth. Therefore, a larger prospective study is required to investigate whether the blood lipid level is related to DR. Further exploration on whether lipid-lowering drugs can help in treating DR is also necessary. This study also found that in elderly men and postmenopausal women, the HbA1c level increased in the BDR and NDR groups. Poor glycemic control could lead to DR progression; however, the difference between groups was not statistically significant and could have been affected by the diabetic duration and other factors. This result was consistent with the studies performed in China and elsewhere [25, 26]. The regression analysis found the diabetic duration as an independent risk factor for DR, which was also consistent with the results of prior epidemiological studies [27]. Therefore, patients with diabetes should pay attention to diet and exercise, actively control their blood glucose levels, and undergo fundus screening and early intervention as soon as possible [28].

However, this study had a few limitations. The sample size needs to be larger to better assess the risk factors for DR in T2DM. Furthermore, the study lacked data on patient's lifestyles and their exposure to the sun. Patients with greater retinal damage might have less exposure to the sun, thus, influencing the results. Future studies should focus more on the patient's lifestyle, especially on the exposure to the sun. Also, this study was a cross-sectional retrospective study and lacked follow-up analysis of the impact of vitamin D on DR, which needs to be further examined in a large, prospective, follow-up study on patients with DR who have been on vitamin D supplementation.

## 8. Conclusions

A close association was found between 25(OH)D and DR in the elderly male patients and postmenopausal women with type 2 diabetes mellitus. In addition, the level of P1NP might be closely related to DR in postmenopausal women with T2DM. Meanwhile, the level of *β*-CTX might be closely related to DR in elderly male patients with T2DM. Clinically, the monitoring of the bone metabolism indices may serve as a new way for predicting the occurrence of DR in T2DM.

## Figures and Tables

**Table 1:** Comparison of general characteristics, biochemical indices, BMD, and bone metabolism indices among the three groups.

	Male patients					Female patients				
Index	NDR (*n* = 268)	BDR (*n* = 60)	PDR (*n* = 24)	*F*	*P*	NDR (*n* = 198)	BDR (*n* = 60)	PDR (*n* = 26)	*F*	*P*
Age (year)	62.64 ± 8.44	62.02 ± 6.51	62.67 ± 8.64	0.15	0.86	63.31 ± 8.06	63.77 ± 8.72	65.65 ± 9.16	0.83	0.40
BMI (kg/m^2^)	25.38 ± 3.32	25.79 ± 3.04	25.44 ± 2.55	0.37	0.69	25.72 ± 3.43	24.92 ± 4.06	26.12 ± 4.32	1.27	0.28
Diabetic duration (year)	10.31 ± 7.53	13.52 ± 7.96^a^	16.67 ± 6.77^a,b^	10.95	<0.05	8.87 ± 7.66	13.87 ± 7.86^a^	14.82 ± 8.61^a,b^	13.96	<0.05
SBP (mmHg)	132.53 ± 16.76	134.82 ± 15.78	140.29 ± 17.50	2.64	0.07	134.60 ± 17.47	138.45 ± 18.36	136.38 ± 18.12	1.12	0.33
DBP (mmHg)	78.35 ± 10.20	77.38 ± 12.05	80.33 ± 7.64	0.70	0.50	76.57 ± 10.23	78.67 ± 10.16	76.88 ± 11.69	0.95	0.39
HbA1c (%)	8.28 ± 1.96	8.39 ± 1.75	8.58 ± 1.38	0.34	0.71	8.55 ± 1.87	8.78 ± 2.07	8.80 ± 1.82	0.42	0.66
FBG (mmol/L)	8.83 ± 4.20	9.13 ± 2.91	8.80 ± 3.37	0.144	0.87	8.43 ± 3.30	9.05 ± 3.68	9.13 ± 3.23	2.20	0.12
TC (mmol/L)	4.11 ± 1.10	3.95 ± 0.96	4.02 ± 0.79	0.42	0.66	4.37 ± 1.01	4.37 ± 1.30	4.92 ± 1.21	2.86	0.06
TG (mmol/L)	1.74 ± 1.24	1.65 ± 0.98	1.61 ± 1.05	0.27	0.76	1.99 ± 1.33	1.85 ± 1.46	2.01 ± 1.37	0.27	0.76
LDL-C (mmol/L)	2.45 ± 0.85	2.51 ± 0.77	2.40 ± 0.73	1.21	0.30	2.56 ± 0.83	2.66 ± 1.04	2.83 ± 1.04	1.05	0.35
HDL-C (mmol/L)	0.98 ± 0.24	0.87 ± 0.19	0.98 ± 0.22	1.13	0.32	1.06 ± 0.33	1.14 ± 0.32	1.08 ± 0.29	1.43	0.24
UA (umol/L)	340.59 ± 86.70	343.40 ± 100.82	355.18 ± 64.64	0.29	0.75	317.55 ± 85.70	317.58 ± 91.12	336.29 ± 103.78	0.49	0.61
eGFR (ml/min/1.73^2^)	91.28 ± 18.02	89.46 ± 18.89	87.50 ± 20.51	0.61	0.54	90.755 ± 17.96	89.35 ± 21.10	82.01 ± 24.62	2.32	0.10
UACR	28.12 ± 50.91	88.43 ± 80.85^a^	101.00 ± 73.01^a,b^	7.18	<0.05	58.32 ± 18.39	252.86 ± 30.14^a^	308.02 ± 86.98^a,b^	6.35	<0.05
PTH	36.68 ± 14.15	38.57 ± 14.66	35.77 ± 11.93	0.50	0.61	37.91 ± 14.34	39.96 ± 15.20	37.23 ± 16.73	0.50	0.61
Lumbar–BMD (g/cm^2^)	0.99 ± 0.16	0.98 ± 0.14	0.97 ± 0.15	0.11	0.90	0.87 ± 0.17	0.86 ± 0.14	0.82 ± 0.20	0.92	0.40
Hip-BMD (g/cm^2^)	0.74 ± 0.11	0.76 ± 0.14	0.77 ± 0.10	1.18	0.31	0.65 ± 0.12	0.66 ± 0.12	0.63 ± 0.17	0.76	0.47
OC (ng/ml)	11.35 ± 4.81	11.24 ± 4.87	10.88 ± 6.73	0.10	0.90	13.96 ± 5.52	13.78 ± 6.08	15.37 ± 8.83	0.69	0.51
*β*-CTX (ng/ml)	0.37 ± 0.25	0.33 ± 0.20^a^	0.26 ± 0.12^a,b^	3.50	<0.05	0.47 ± 0.61	0.51 ± 0.78	0.47 ± 0.34	0.06	0.94
P1NP (ng/ml)	34.70 ± 14.36	34.89 ± 13.18	32.68 ± 15.75	0.24	0.79	44.21 ± 19.27	48.18 ± 23.05^a^	57.34 ± 31.57^a,b^	3.44	<0.05
25(OH)D (ng/ml)	17.37 ± 7.43	15.57 ± 6.22^a^	12.59 ± 6.37^a,b^	4.93	<0.05	14.69 ± 5.69	13.35 ± 5.11^a^	11.27 ± 5.05^a,b^	4.30	<0.05

Note: ^a^*P* < 0.05 compared with the NDR group; ^b^*P* < 0.05 compared with the BDR group. Abbreviations: *β*-CTX: beta C-terminal telopeptide; BMD: bone mineral density; BMI: body mass index; Ca: calcium; DBP: diastolic blood pressure; eGFR: glomerular filtration rate; FBG: fasting blood glucose; HbA1c: glycosylated hemoglobin; HDL-C: high-density lipoprotein cholesterol; LDL-C: low-density lipoprotein cholesterol; OC: osteocalcin; 25(OH)D: 25-hydroxyvitamin D; P1NP: procollagen 1 intact N-terminal; PTH: parathyroid hormone; SBP: systolic blood pressure; TC: total cholesterol; TG: triglyceride; UA: uric acid; UACR: urinary albumin to creatinine ratios.

**Table 2:** Relationship between vitamin D status and DR.

	Male patients					Female patients				
	NDR (*n* = 268)	BDR (*n* = 60)	PDR (*n* = 24)	*χ* ^2^	*P*	NDR (*n* = 198)	BDR (*n* = 60)	PDR (*n* = 26)	*χ* ^2^	*P*
Vitamin D deficiency (%)	173 (64.6%)	46 (76.6%)	21 (87.5%)			123 (62.1%)	45 (75.0%)	22 (84.6%)		
Nonvitamin D deficiency (%)	95 (35.4%)	14 (23.4%)	3 (12.5%)	7.75	<0.05	75 (37.9%)	15 (25.0%)	4 (15.4%)	7.51	<0.05

**Table 3:** Logistic regression analysis of the risk factors for DR in elderly male patients and postmenopausal women with T2DM.

DR							
Elderly men (*n* = 352)				Postmenopausal women (*n* = 284)			
Index	*Β*st	OR (95CI%)	*P*	Index	*Β*st	OR (95CI%)	*P*
Model 1				Model 1			
DM duration	0.07	1.07 (1.04, 1.11)	<0.05	DM duration	0.83	1.09 (1.05, 1.12)	<0.05
UACR	0.04	1.00 (1.00, 1.01)	<0.05	UACR	0.01	1.03 (1.00, 1.07)	<0.05
*β*-CTX	-1.71	0.18 (0.04, 0.76)	<0.05	P1NP	0.01	1.01 (1.00, 1.03)	<0.05
25(OH)D	-0.05	0.95 (0.91, 0.99)	<0.05	25(OH)D	-0.07	0.94 (0.89, 0.99)	<0.05

Model 2 (adjusted)				Model 2 (adjusted)			
DM duration	0.06	1.06 (1.00, 1.11)	<0.05	DM duration	0.10	1.11 (1.05, 1.17)	<0.05
UACR	0.01	1.01 (1.00, 1.01)	<0.05	UACR	0.00	1.01 (1.00, 1.02)	<0.05
*β*-CTX	-1.57	0.21 (0.03, 1.48)	0.12	P1NP	0.00	1.00 (0.98, 1.03)	0.72
25(OH)D	-0.01	0.99 (0.99, 1.00)	<0.05	25(OH)D	-0.08	0.92 (0.85, 0.98)	<0.05

Model 2 adjusted for age, BMI, duration, BP, blood lipid levels, blood glucose, PTH, and eGFR.

**Table 4:** Logistic regression analysis of the risk factors for PDR in patients with DR.

PDR							
Elderly men (*n* = 352)				Postmenopausal women (*n* = 284)			
Index	*Β*st	OR (95CI%)	*P*	Index	*Β*st	OR (95CI%)	*P*
Model 1				Model 1			
DM duration	0.05	1.06 (1.00, 1.13)	<0.05	DM duration	0.13	1.17 (1.02, 1.33)	<0.05
UACR	0.00	1.00 (0.99, 1.00)	0.85	UACR	0.07	1.02 (1.01, 1.04)	<0.05
*β*-CTX	-2.90	0.06 (0.01, 2.10)	0.12	P1NP	0.01	1.01 (0.97, 1.03)	0.32
25(OH)D	-0.09	0.92 (0.83, 0.99)	<0.05	25(OH)D	-0.06	0.90 (0.82, 0.97)	<0.05

Model 2 (adjusted)				Model 2 (adjusted)			
DM duration	0.04	1.03 (1.01, 1.06)	<0.05	DM duration	0.10	1.13 (1.02, 1.23)	<0.05
UACR	-0.01	0.99 (0.97, 1.01)	0.39	UACR	0.01	1.05 (0.87, 1.15)	0.45
*β*-CTX	-3.58	0.73 (0.34, 1.65)	0.43	P1NP	0.01	1.01 (0.97, 1.03)	0.32
25(OH)D	-1.00	0.91 (0.76, 1.06)	0.21	25(OH)D	-0.02	0.87 (0.77, 1.28)	0.29

Model 2 adjusted for age, BMI, duration, BP, blood lipid level, blood glucose level, PTH, and eGFR.

## Data Availability

The data used to support the findings of this study are available from the corresponding author upon request.
